# PKCζ Promotes Breast Cancer Invasion by Regulating Expression of E-cadherin and Zonula Occludens-1 (ZO-1) via NFκB-p65

**DOI:** 10.1038/srep12520

**Published:** 2015-07-28

**Authors:** Arindam Paul, Marsha Danley, Biswarup Saha, Ossama Tawfik, Soumen Paul

**Affiliations:** 1 The University of Kansas Cancer Center, University of Kansas Medical Center, Kansas City, KS 66160, USA; 2 Department of Pathology and Laboratory Medicine, University of Kansas Medical Center, Kansas City, KS 66160, USA; 3 Institute of Reproductive Health & Regenerative Medicine, University of Kansas Medical Center, Kansas City, KS 66160, USA

## Abstract

Atypical Protein Kinase C zeta (PKCζ) forms Partitioning-defective (PAR) polarity complex for apico-basal distribution of membrane proteins essential to maintain normal cellular junctional complexes and tissue homeostasis. Consistently, tumor suppressive role of PKCζ has been established for multiple human cancers. However, recent studies also indicate pro-oncogenic function of PKCζ without firm understanding of detailed molecular mechanism. Here we report a possible mechanism of oncogenic PKCζ signaling in the context of breast cancer. We observed that depletion of PKCζ promotes epithelial morphology in mesenchymal-like MDA-MB-231 cells. The induction of epithelial morphology is associated with significant upregulation of adherens junction (AJ) protein E-cadherin and tight junction (TJ) protein Zonula Occludens-1 (ZO-1). Functionally, depletion of PKCζ significantly inhibits invasion and metastatic progression. Consistently, we observed higher expression and activation of PKCζ signaling in invasive and metastatic breast cancers compared to non-invasive diseases. Mechanistically, an oncogenic PKCζ– NFκB-p65 signaling node might be involved to suppress E-cadherin and ZO-1 expression and ectopic expression of a constitutively active form of NFκB-p65 (S536E-NFκB-p65) significantly rescues invasive potential of PKCζ-depleted breast cancer cells. Thus, our study discovered a PKCζ - NFκB-p65 signaling pathway might be involved to alter cellular junctional dynamics for breast cancer invasive progression.

Breast cancer is one of the leading causes of cancer related death in women worldwide^1^. Clinically, breast cancer is considered as a heterogeneous disease and heterogeneity of breast cancer disease provides a great challenge for developing successful therapy. Comprehensive gene expression profiling indicated at least three major subtypes of breast cancer – luminal, HER2-positive, and basal-like^2^,^3^,^4^,^5^. These subtypes of breast cancer are significantly different in clinical characteristics such as associated risk factors, preferable sites of metastasis, and expression of targetable surface receptors such as estrogen receptor (ER), progesterone receptor (PR), and epidermal growth factor receptor 2 (ERBB2/HER2)^6^. While the luminal (ER/PR positive) and the HER2-positive (with amplified HER2 expression) breast cancer patients could be benefited from endocrine and HER2-targeted therapies^7^, chemotherapy is the only therapeutic option currently available for basal-like (also called triple negative breast cancers or TNBC, no expression of ER, PR, and HER2)^8^ breast tumors.

During invasive progression, breast cancer cells undergo sequential developmental alterations and eventually acquire the capacity to form metastatic growth for tumor recurrence^9^,^10^,^11^. Similar to most other cancers, metastases are also considered as major reason for breast cancer-related deaths^9^,^12^,^13^,^14^ and development of recurrence/metastases can occur even after the initial successful therapeutic responses^15^. Thus, breast cancer patients are always at risk to develop recurrence/metastasis throughout their life^15^. As a result, identification of signaling pathways to inhibit invasive and metastatic properties of breast cancer cells is always critical for the development of successful therapies.

Invasive progression of breast cancer is initiated through the process called epithelial-to-mesenchymal transition (EMT), a developmental switch well known for tissue remodeling during normal embryonic development^11^,^16^,^17^. The reverse process of EMT is known as mesenchymal-to-epithelial transition (MET) and characterized by the transition of mesenchymal cells to acquire epithelial characteristics^18^. During EMT, polarized epithelial cells transform to a highly motile mesenchymal phenotype with rearranged cytoskeleton via the loss of cell polarity. Intercellular junctions such as adherens junctions (AJ), tight junctions (TJ), gap junctions, and desmosomes are responsible to maintain cell polarity in epithelial tissues and these intercellular junctions are disrupted during the process of EMT^17^,^18^,^19^. Highly conserved polarity proteins including the members of the PAR polarity complex regulate proper distributions of these cellular junctional complexes in the plasma membrane^20^,^21^. The PAR polarity complexes contain PAR3, PAR6, and aPKC isozymes PKCζ and PKCλ/ι and activation of aPKC signaling is essential for establishing functional PAR polarity complexes at the apical-lateral border in epithelial cells^22^,^23^. In vertebrate epithelial cells, apical-lateral border is structurally defined by TJs, which prevents diffusion of the membrane proteins to ensure apical and basal polarity^24^,^25^,^26^. Failure to maintain correct apico-basal polarity due to disruption of PAR polarity complex or down-regulation of polarity and/or junctional proteins are implicated in promoting EMT and tissue infiltration of breast and other cancers of epithelial origin^20^,^27^,^28^,^29^,^30^.

Atypical PKCs, PKCζ and PKCλ/ι, are the member of PKC family of serine/threonine kinases, which are involved in multiple signal transduction pathways. Activation of aPKCs is independent of both Ca^2+^ and diacylglycerol compared to conventional PKC (cPKCs; PKCα, PKCβI, PKCβII, and PKCγ) and novel PKC (nPKCs; PKCδ, PKCε, PKCη, and PKCθ) subfamilies. The conventional PKC members are activated by diacylglycerol and Ca^2+^-dependent phospholipid binding to their conserved domains and the novel PKC members are activated only by diacylglycerol and phospholipids, but independent of Ca^2+^ ion^31^,^32^. Although aPKC molecules play a central role to maintain epithelial cell polarity, multiple studies showed that aPKC signaling often induce invasion and metaspromotes breast cancer invasive progressiong breast cancer^33^,^34^,^35^,^36^,^37^,^38^,^39^,^40^,^41^,^42^. Recently, we and other laboratories showed that aPKC isozyme, PKCλ/ι promotes breast cancer invasive progression^33^,^37^,^43^,^44^. On the other hand, the other aPKC isozyme, PKCζ, has both tumor suppressive and tumor promoting functions including for breast cancer development^45^,^46^,^47^,^48^,^49^,^50^,^51^,^52^. The PKCζ found to be one of the frequently mutated genes associated with TNBC^53^ and reported to regulate proliferation and chemokine-triggered migration of breast cancer cells^42^,^54^,^55^,^56^,^57^,^58^. In contrast, over-expression of PKCζ also showed growth inhibition of human MDA-MB-468 breast cancer cells^59^. However, these cellular functions of PKCζ are concluded based on the use of non-specific small molecule inhibitors and/or pseudo-substrate peptides^60^ and overall, the molecular mechanisms of PKCζ-mediated regulation of breast cancer disease progression are largely unknown.

Here, we investigated the role of PKCζ in breast cancer development. We found that PKCζ signaling is highly active in invasive and metastatic breast cancers compared to non-invasive ductal carcinoma *in situ* (DCIS) and depletion of PKCζ inhibits invasion and metastasis of breast cancer cells in experimental animal models. Interestingly, we observed that loss of PKCζ promotes MET in highly metastatic, mesenchymal-like MDA-MB-231 cells with induction of cell-cell adhesion. Our molecular analyses indicate that depletion of PKCζ inhibits nuclear localization of NFκB-p65 leading to elevated expressions of epithelial cell specific adherens junction protein E-cadherin and tight junction protein ZO1. We also found that ectopic expression of a constitutively active form of NFκB-p65 (S536E-NFκB-p65) significantly rescues invasive potential of PKCζ-depleted breast cancer cells. Collectively, our results provide evidence for an oncogenic PKCζ–NFκB-p65 signaling node that suppresses E-cadherin and ZO-1 expression in breast cancer cells and might promote EMT to facilitate *in situ* to invasive transition of breast cancers.

## Results

### PKCζ Regulates Cell-Cell Adhesion in the Absence of Functional PAR Polarity Complex

**Figure 1:**
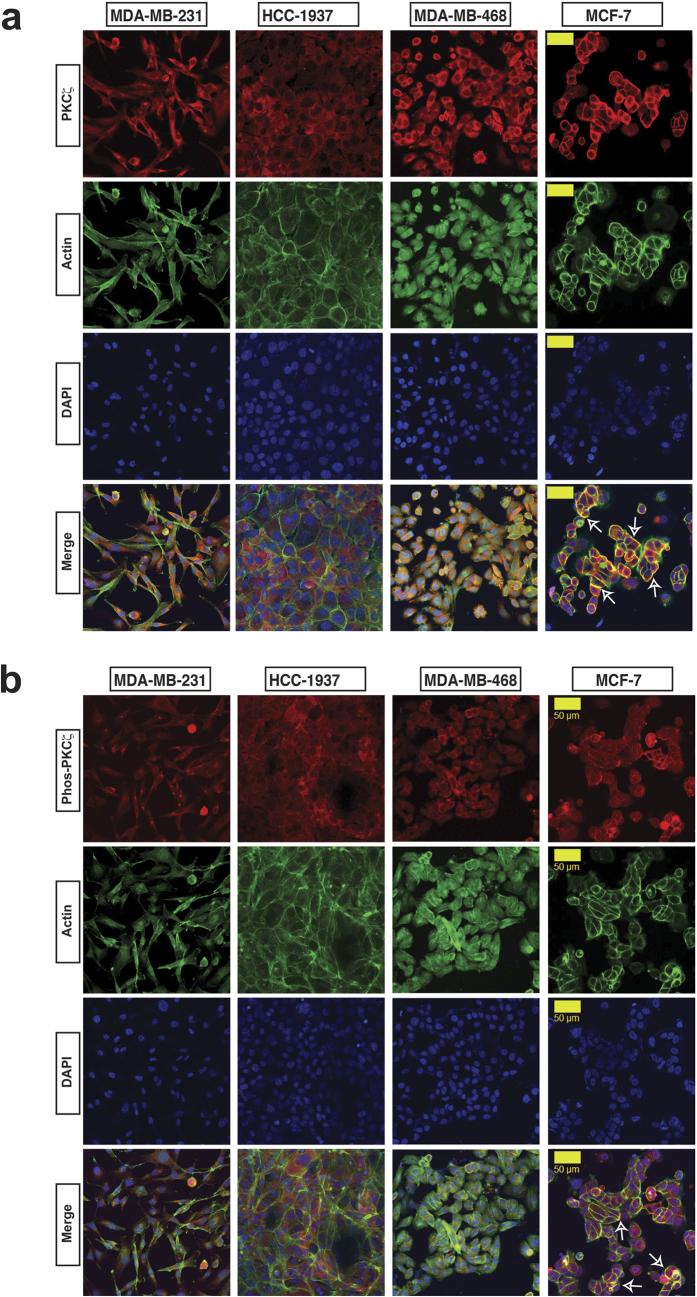
PKCζ Signaling in Breast Cancer Cells. Expression of PKCζ (**a**) and phospho-PKCζ (**b**) in basal-like MDA-MB-231, HCC-1937, and MDA-MB-468 cells in comparison with luminal MCF-7 cells. Expression of PKCζ and phospho-PKCζ showed in red, actin in green and nuclear staining showed by DAPI. Yellow scale bar 50 μM. White arrows indicated localization of PKCζ and phospho-PKCζ at the plasma membrane domains of MCF-7 cells.

In epithelial cells, PKCζ serves as an effector of the conserved PAR polarity complex, which is located at the plasma membrane domains for the regulation of apical-basal polarity by stimulating biogenesis of cell-cell junctions^61^,^62^. Thus, to investigate PKCζ signaling in breast cancer, we have tested expression and localization of PKCζ in multiple human breast cancer cell lines including luminal MCF-7 and three basal-like cells such as MDA-MB-231, MDA-MB-468, and HCC-1937^19^,^63^,^64^,^65^,^66^,^67^. PKCζ is abundantly expressed in all cell lines (Supplementary Figure S1a). However, we noticed differential PKCζ localization patterns. In basal-like cells, expression of PKCζ indicated a diffused localization pattern without any prominent distribution at the plasma membrane domains (Fig. 1a). On the other hand, expression of PKCζ in luminal MCF-7 cells was observed predominantly at the plasma membrane domains (Fig. 1a) and consistent with formation of PAR polarity complex as reported earlier^20^,^24^,^26^,^30^,^49^. Interestingly, phospho-PKCζ (phosphorylated at T410) expression also showed similar trends (Fig. 1b). Since phosphorylation at T-410 is essential for kinase activity of PKCζ^49^,^68^, our observations indicate the presence of active PKCζ signaling in all tested breast cancer cells. However, the absence of PKCζ and phospho-PKCζ in the plasma membrane of basal-like cells indicate the absence of PKCζ-containing PAR polarity complexes (Fig. 1a,b).

Comprehensive gene expression analyses indicate that MDA-MB-231 cells posses a mesenchymal-like phenotype with more stromal-like/fibroblastic character without common epithelial gene set^19^,^67^. So, we predicted that functional PAR polarity complexes are absent in MDA-MB-231 cells. To confirm, we investigated expression and localization of PAR3 and PAR6. Interestingly, both PAR3 and PAR6 are expressed in MDA-MB-231 cells, however, they showed diffused expression patterns with localization both in the cytoplasm and the nuclei (Supplementary Figure S1b). The absence of localization of PKCζ, phospho-PKCζ, PAR3, and PAR6 at the plasma membrane or at any prominent apical/basal domains of MDA-MB-231 cells strongly supports the absence of PAR polarity complexes.

**Figure 2:**
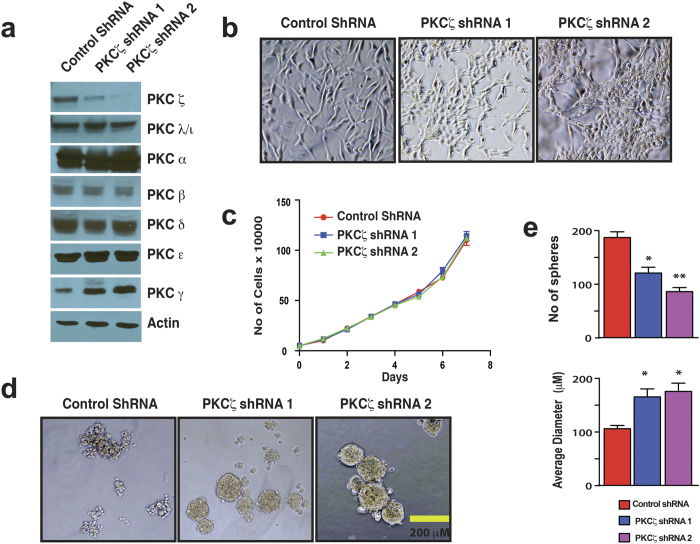
PKCζ Signaling Regulates Cell-Cell Adhesion in Breast Cancer Cells. (**a**) Western blot analysis indicating specific knockdown of PKCζ in MDA-MB-231 cells. (**b**) Morphologies of MDA-MB-231 cell with and without PKCζ depletion. (**c**) Specific depletion of PKCζ has no effect on cell proliferation. (**d**) Morphologies of MDA-MB-231 cell aggregates with and without PKCζ depletion. (**e**) Quantification of number of MDA-MB-231 cell aggregates and their size with and without PKCζ depletion. Results represent means ± S.E.M. *P* values were calculated one-way ANOVA with Bonferroni post-test. *P values ≤ 0.01, **P values ≤ 0.001, ***P values ≤ 0.0001.

Based on the expression and cellular localization patterns in the highly invasive mesenchymal-like MDA-MB-231 and other basal-like cells, we hypothesized that PKCζ might mediate its function independent of PAR polarity complex. Therefore, we specifically depleted PKCζ in MDA-MB-231 cells via RNA interference (RNAi) (Fig. 2a). Depletion of PKCζ in MDA-MB-231 cells induced a dramatic morphological alteration and organized them into a highly clustered morphology from scattered, fibroblast-like culture without any effect in cell proliferation rates (Fig. 2b,c). These alterations in morphology indicate more cell-cell contact formation. For confirmation, we tested PKCζ-depleted cells for conventional cell aggregation assay to access cell-cell junction formation^69^. We observed that depletion of PKCζ transformed the appearance of MDA-MB-231 cell aggregates towards more smooth and round-shaped compared to control (Fig. 2d). We also found that PKCζ-depleted cells generated less number of cell aggregates, however, the cell aggregates were significantly larger compared to control indicating more intercellular integrity (Fig. 2e). Overall, these results indicate that depletion of PKCζ induces cell-cell adhesion in MDA-MB-231 cells.

### Depletion of PKCζ Induces Expression of E-cadherin and ZO-1 and Prevents Invasive Potential

**Figure 3:**
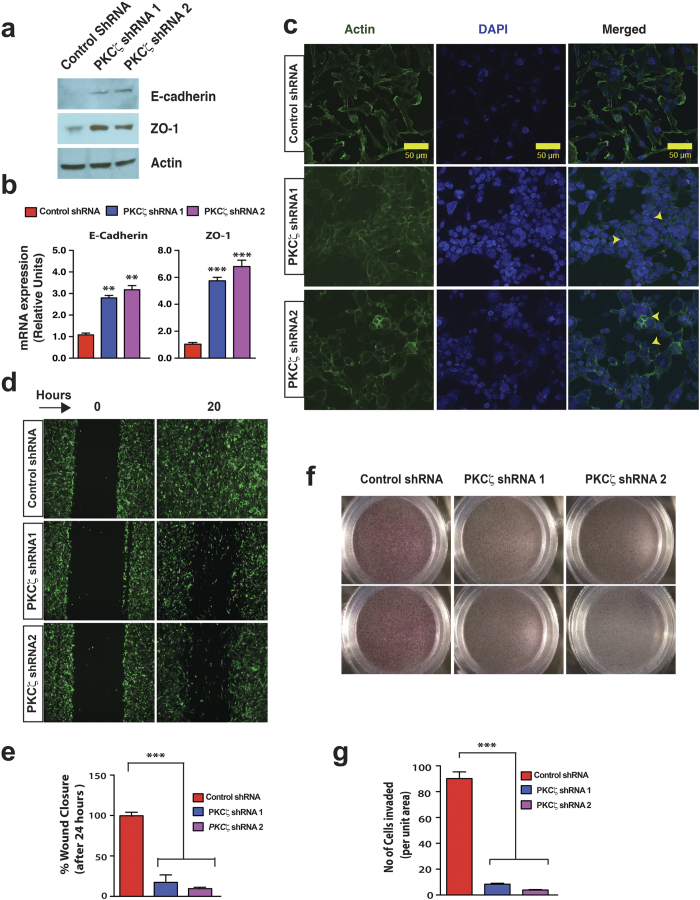
PKCζ Signaling Regulates Cell-Cell Junction Dynamics and Invasion. (**a**) Western blot analysis of E-cadherin and ZO-1 after specific knockdown of PKCζ in MDA-MB-231 cells. (**b**) Quantitative RT-PCR measurements of E-cadherin and ZO-1 after specific knockdown of PKCζ in MDA-MB-231 cells. Results represent means ± S.E.M. *P* values were calculated by two-tailed unpaired Student’s *t* test. **P values ≤ 0.01, ***P values ≤ 0.001. (**c**) Rearrangement of actin in MDA-MB-231 cells with and without PKCζ depletion. Specific knockdown of PKCζ in MDA-MB-231 cells induced appearance of cortical actin showed by yellow arrowheads. Scale bar 50 μM. (**d**) Wound closure assays of PKCζ-depleted MDA-MB-231 cells. (**e**) Quantification of wound closure assays (*n* = 3). (**f**) Transwell invasion of PKCζ-depleted MDA-MB-231 cells. (**g**) Quantification of invasion assays (each field was divided into 9 unit areas and 3 fields per condition). For all quantifications, results represent means ± S.E.M. *P* values were calculated by two-tailed unpaired Student’s *t* test. ***P values ≤ 0.001.

In epithelial tissues, cell-cell contacts are regulated by formation of several intercellular junctions including AJs and TJs. Thus, we tested PKCζ-depleted MDA-MB-231 cells for expression analysis of two junctional proteins - E-cadherin, localized at AJ^17^,^70^ and ZO-1, localized at TJ to link TJ and cytoskeleton^71^. Our analyses indicate that the morphological changes of PKCζ-depleted MDA-MB-231 cells were indeed associated with upregulation of both E-cadherin and ZO-1 (Fig. 3a,b). We also tested expression of other TJ and AJ proteins such as ZO-2, ZO-3, and Afadin via western blot analysis, but did not observe any significant changes at protein levels (data not shown). Since E-cadherin and ZO-1 are considered as the epithelial markers^72^, we further tested formation of cortical actin in PKCζ-depleted cells. Immunofluorescence staining revealed formation of cortical actin in PKCζ-depleted cells (Fig. 3c) confirming cytoskeletal rearrangement consistent with epithelial characteristics^19^. Expression analysis of PAR3 in PKCζ-depleted cells showed punctate appearance throughout the cells whereas PAR6 expression was restricted within the cytoplasm indicating the absence of functional PAR polarity complexes (Supplementary Figure S2a & S2b). These results strongly indicate that depletion of PKCζ in MDA-MB-231 cells induce MET-like process and promotes epithelial morphology in the absence of functional PAR polarity complexes.

Since the reverse process of MET i.e. EMT is implicated in conferring invasive potential^14^, we tested whether induction of epithelial characteristics in PKCζ-depleted MDA-MB-231 cells is associated with loss of invasiveness. We found that depletion of PKCζ significantly inhibited invasive potential of MDA-MB-231 cells when tested via wound closure assays (Fig. 3d,e) and matrigel-coated transwell invasion assays (Fig. 3f,g).

### Depletion of PKCζ Inhibits Breast Cancer Metastasis

**Figure 4:**
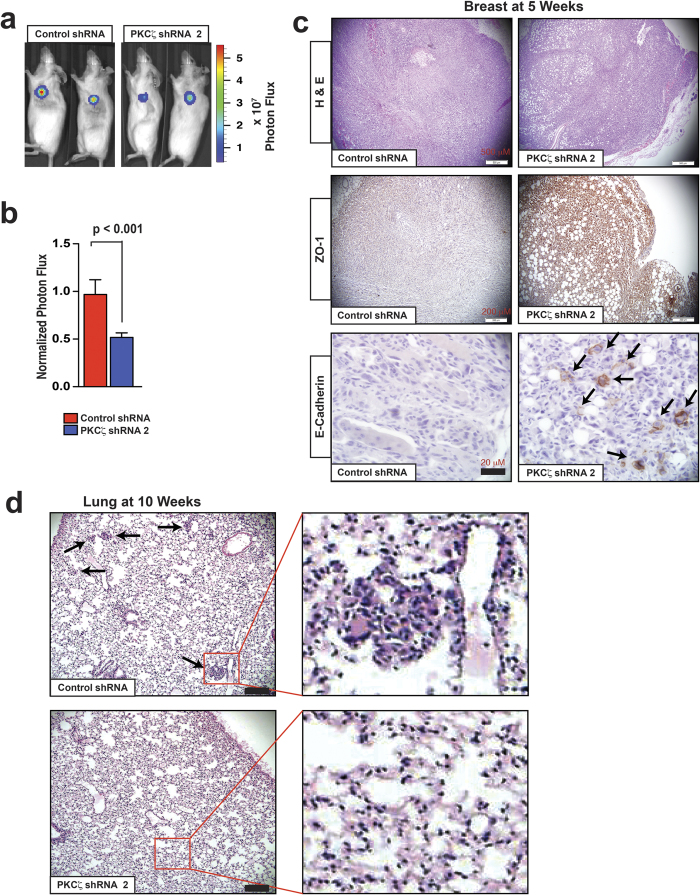
Depletion of PKCζ Inhibits Breast Cancer Metastasis. (**a**) Representative whole-animal images at five weeks after orthotopic transplantation of MDA-MB-231-luc cells with and without PKCζ depletion. (**b**) Quantification of tumor growth via luminescence measurements at five weeks after orthotopic transplantation (*n* = 8). Results represent means ± S.E.M. *P* values were calculated one-way ANOVA with Bonferroni post-test. **(c)** H&E and immunohistochemical analysis of ZO-1 and E-cadherin expression in xenograft breast tumors removed at five weeks after orthotopic transplantation. The mice were kept alive for another five weeks for spontaneous metastasis to lung. Black arrows indicate expression of E-cadherin. (**d**) H&E staining of lung tissues at 10 weeks after orthotopic transplantation. Black arrows showed lung colonization and outlined areas by indicated red squares represent the higher magnification images in right panels.

Next, we tested *in vivo* functional importance of PKCζ signaling in breast cancer using experimental animal models utilizing MDA-MB-231 cells expressing a luciferase reporter (MDA-MB-231-luc)^33^. We selected shRNA clone no 2 to deplete PKCζ in MDA-MB-231-luc cells for better knockdown efficiency (Fig. 2a). We transplanted MDA-MB-231-luc cells with or without PKCζ-depletion orthotopically into the 2^nd^ mammary glands of immunodeficient mice, removed primary tumor at five weeks, and observed for spontaneous metastasis at lung for another five weeks^33^. We observed that the orthotopic tumors formed by PKCζ-depleted cells showed nearly 50% reduced primary tumor growth at five weeks compared to control (Fig. 4a,b). Notably, we observed higher expression of ZO-1 and E-cadherin in PKCζ-depleted xenograft tumors compared to control (Fig. 4c). Both PAR3 and PAR6 polarity proteins showed diffuse expression patterns in the PKCζ-depleted tumors (Supplementary Figure S3).

**Figure 5:**
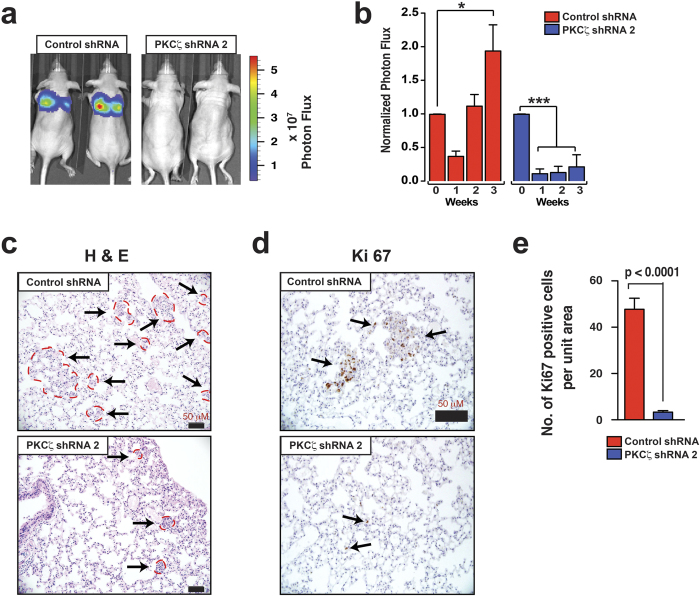
Depletion of PKCζ Inhibits Lung Metastatic Colonization. (**a**) Representative whole-animal images at three weeks after intravenous transplantation (via tail vein) of MDA-MB-231-luc cells with and without PKCζ depletion. (**b**) Quantification of metastatic lung colonization via luminescence measurements (*n* = 5). Results represent means ± S.E.M. *P* values were calculated using one-way ANOVA with Bonferroni post-test. *P values ≤ 0.01, **P values ≤ 0.001, ***P values ≤ 0.0001. (**c**) H&E staining of lung tissues three weeks after intravenous transplantation. Indicated regions by perforated red lines and arrows showed lung colonization. Scale bar 50 μM. **(d)** Ki-67 staining of lung tissues at three weeks post-transplantation. Scale bar 50 μM. (**e**) Quantification of Ki67 staining (*n* = 12). Results represent means ± S.E.M. *P* values were calculated by one-way ANOVA with Bonferroni post-test.

Next, we searched for spontaneous lung metastasis five weeks after resection of the primary tumors (i.e. at ten weeks after orthotopic transplantation). We observed lung metastasis in mice transplanted with control MDA-MB-231 cells at ten weeks as reported previously^33^. However, no metastatic event was observed in mice transplanted with PKCζ-depleted MDA-MB-231 cells in that time frame (Fig. 4d). To rule out the possibility that the reduced tumor growth of PKCζ-depleted cells might be the reason for the lack of lung metastasis, we further tested metastatic potential via lung colonization after intravenous transplantation^33^. We have transplanted both control and PKCζ-depleted MDA-MB-231-luc cells via tail vein and monitored lung colonization for three weeks via bioluminescent imaging (Fig. 5a). We observed dramatic inhibition in lung metastatic colonization with the PKCζ-depleted cells (Fig. 5b–e). These observations indicate that PKCζ signaling is critical for breast cancer metastasis *in vivo*.

### Human breast cancer shows highly active PKCζ signaling

**Figure 6:**
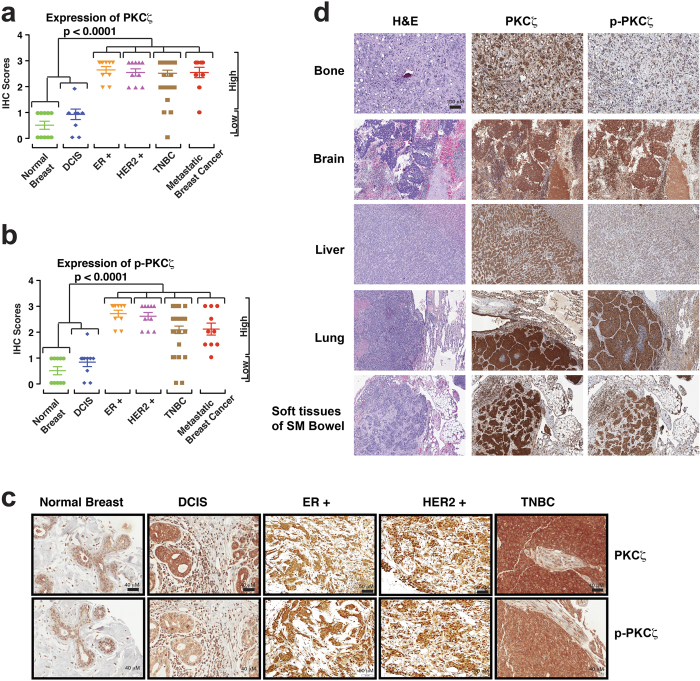
Aggressive Breast Cancers Are Associated With Higher Expression and Activation of PKCζ. Expression of PKCζ (**a**) and phospho-PKCζ (**b**) in human normal breast, DCIS, IDCs (ER+, HER2+, and TNBC), and metastatic breast cancer samples. Results were expressed as IHC scores of individual samples (by two independent pathologists) using a scale 0 to 3. IHC scores in between 0 to 1 considered as low expression whereas IHC scores >1 considered as high expression. Majority of IDCs and metastatic breast cancer samples showed high expression of PKCζ and phospho-PKCζ. *P*-values were calculated by two-way ANOVA with Bonferroni post-test. (**c**) Representative images showing expression and localization of PKCζ and phospho-PKCζ in normal breast, DCIS, and IDCs with ER+, HER2+, and triple negative status. Expression of PKCζ and phospho-PKCζ gradually increased from normal breast to DCIS, and significantly increased in IDC and metastatic breast cancer samples. (**d**) Expression of PKCζ and phospho-PKCζ in human metastatic breast cancers. Scale bar 100 μM.

Prompted by our *in vitro* and *in vivo* observations, we sought to investigate PKCζ signaling in human breast cancer samples. Previous reports indicated conflicting findings to correlate PKCζ expression with breast cancer clinico-pathological characteristics. Studies by *Whyte et al.*^49^ indicated that low levels of PKCζ mRNA expression are more significantly associated with poor clinical outcome of breast cancer patients including for the ‘poorly differentiated’ tumors. On the other hand, studies by *Yin et al.*^50^ indicated higher expression of PKCζ associated with advanced clinical stages of breast cancer including larger tumor size, lymph node metastasis, and poor survival rates. For confirmation, we tested expression of both PKCζ and phospho-PKCζ in a cohort of human breast cancer samples consisting of normal breasts, ductal carcinoma *in situ* (DCIS), invasive ductal carcinomas (IDCs) of ER-positive, HER2-positive, and TNBC subtypes, and metastatic breast cancers (Fig. 6a,b). Immunohistochemical analyses showed that expression levels of PKCζ were significantly higher in IDCs and metastatic breast cancers compared to normal breast and non-invasive DCIS samples (Fig. 6a,c,d). Notably, expression of phospho-PKCζ (phosphorylated at T410), indicative of active PKCζ signaling, also showed similar trends (Fig. 6b–d). These observations indicate that higher expression and activation of PKCζ signaling significantly associated with invasive progression of breast cancer. Interestingly, the expression patterns of both PKCζ and phospho-PKCζ in TNBC and metastatic breast cancer samples are mainly cytoplasmic with occasional nuclear staining similar to the immunostaining patterns as observed in xenograft tumor formed by MDA-MB-231 cells (Supplementary Figure S4) and consistent with pro-oncogenic role of PKCζ signaling in breast cancer.

### PKCζ regulates NFκB-p65 (RelA) Nuclear Translocation and Transcriptional Activity to Regulate Expression of E-Cadherin and ZO-1

**Figure 7:**
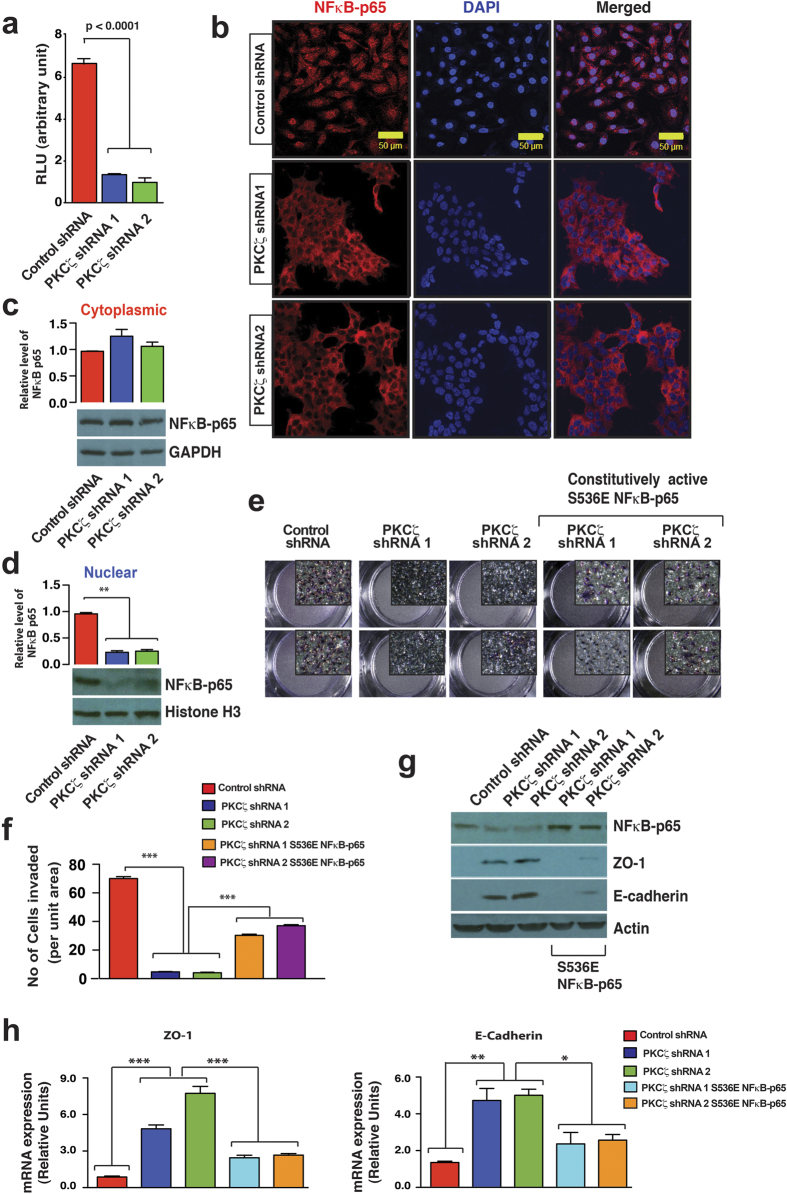
Involvement of PKCζ-NFκB Regulatory Axis. (**a**) NFκB reporter gene assay of MDA-MB-231 cells with and without PKCζ depletion (*n* = 3). Results represent means ± S.E.M. *P* values were calculated using two-tailed unpaired Student’s *t* test. (**b**) Localization of NFκB-p65 in MDA-MB-231 cells with and without PKCζ depletion. Expression of NFκB-p65 showed in red and nuclear staining showed by DAPI. Scale bar, 50 μM. Western blot analysis of cytolasmic NFκB-p65 (**c**) and nuclear NFκB-p65 (**d**) expression level after PKCζ depletion indicating impaired NFκB-p65 nuclear translocation. For quantification, results represent means ± S.E.M. (*n* = 3) and *P* value was calculated using one-way ANOVA with Bonferroni post-test. (**e**) Rescue of invasive potential in PKCζ-depleted MDA-MB-231 cells via ectopic expression of constitutively active S536E NFκB-p65 mutant. Inset represents higher magnification image of the corresponding transwell filter. (**f**) Quantification of invasion assay performed by counting cells present per unit area (each field was divided into 9 unit areas and 3 fields per condition) indicating significant rescue of invasion. For all quantifications, results represent means ± S.E.M. (*n* = 3). *P* values were calculated by one-way ANOVA with Bonferroni post-test. *P values ≤ 0.05, **P values ≤ 0.01, ***P values ≤ 0.001. (**g**) Western blot analysis of NFκB-p65, ZO-1, E-cadherin, and Actin after ectopic expression of a constitutively active S536E NFκB-p65 mutant in PKCζ-depleted MDA-MB-231 cells. (**h**) Quantitative RT-PCR measurements of ZO-1 and E-cadherin after ectopic expression of a constitutively active S536E NFκB-p65 mutant in PKCζ-depleted MDA-MB-231 cells. Results represent means ± S.E.M. *P* values were calculated by two-tailed unpaired Student’s *t* test. *P values ≤ 0.05, **P values ≤ 0.01, ***P values ≤ 0.001.

To understand the mechanism of PKCζ signaling mediated regulation of breast cancer progression in the absence of functional PAR complex, we searched for transcription factors known to be modulated by PKCζ signaling. We hypothesized that the transcription factor(s), regulated by PKCζ signaling, might link how depletion of PKCζ resulted in significant increase of E-cadherin and ZO-1 expression. In multiple cell types, PKCζ signaling has been shown to regulate NFκB-p65 (RelA) transcriptional activity^73^,^74^,^75^. In addition, NFκB-p65 is known to repress expression of both E-Cadherin and ZO1^76^,^77^. Thus, to understand the possible molecular mechanism of PKCζ-mediated regulation of human breast cancer development, we focused on NFκB-p65. We performed NFκB reporter gene analysis and observed repression of endogenous NFκB transcription activity in the PKCζ-depleted MDA-MB-231 cells compared to control (Fig. 7a). Furthermore, we observed a dramatic reduction of NFκB-p65 nuclear localization (Fig. 7b–d). Importantly, depletion of PKCζ in HCC-1937 cells also showed repressed NFκB transcription activity and impaired nuclear localization of NFκB-p65 (Supplementary Figure S5a-S5c). For further confirmation, we tested invasive potential of PKCζ-depleted MDA-MB-231 cells after ectopic expression of a constitutively active S536E RelA construct^78^. We observed significant rescue of invasive phenotype as well as repressed expression of both ZO-1 and E-cadherin in PKCζ-depleted MDA-MB-231 cells (Fig. 7e–h). These results indicate that PKCζ-NFκB signaling node certainly linked to regulate breast cancer progression by regulating expression of cellular junctional proteins such as E-cadherin and ZO-1.

## Discussion

Functional importance of PKCζ in epithelial cells is often assigned to the establishment of a PAR polarity complex, which has been implicated in breast cancer metastasis^29^,^79^,^80^. Interestingly, our findings in this study indicate that a PAR polarity complex-independent function of PKCζ contributes to invasive progression of breast cancer. Depletion of PKCζ induced intercellular adhesion of mesenchymal-like MDA-MB-231 cells and transformed them toward epithelial phenotypes in the absence of a functional PAR polarity complex. Importantly, depletion of PKCζ significantly induced expression of junctional proteins E-cadherin and ZO-1. Downregulation of both E-cadherin and ZO-1 are associated with cancer progression including breast cancer^81^,^82^. Consistently, both *in vitro* as well as *in vivo* functional assays indicate that depletion of PKCζ inhibits invasive potential of mesenchymal-like MDA-MB-231 cells and significantly reduce breast tumor metastasis. Our study with human patients samples further supports the importance of PKCζ during invasive progression of breast cancer. Expression and phosphorylation analyses confirmed that aggressive forms of breast cancers i.e. IDCs and metastatic breast cancers are associated with higher expression and activation of PKCζ. Thus, our results indicate an oncogenic PKCζ signaling node involved in breast cancer invasion and metastasis.

Clinical relevance of PKCζ expression and enzymatic activity in different human cancers has been reported previously^45^,^46^,^47^,^48^,^49^,^50^. However, both up- and down-regulation of PKCζ were observed in human cancers indicating tissue specific role of this enzyme as an oncogene or as a tumor suppressor^45^,^46^,^47^,^48^,^49^,^50^,^51^. In fact, conflicting findings of *Whyte et al.*^49^ and *Yin et al.*^50^ in breast cancer also indicated that function of PKCζ is context dependent and thus, detailed research is required to dissect molecular mechanism for these two opposite functions. Previous reports indicate that PKCζ can directly phosphorylate S311 residue of NFκB-p65 to regulate transcription activity and this mechanism is associated with stress included metabolic reprogramming where PKCζ acts as a tumor suppressor^45^,^47^. On the other hand, our results indicate a possible mechanism by which PKCζ can function as an oncogene. We found that PKCζ signaling regulates cell-cell junctional dynamics also through an NFκB-p65-related mechanism and phosphorylation of S536 residue of NFκB-p65 is involved in this process.

Constitutive NFκB activity is often involved in proliferation of basal-like breast cancer cells^83^,^84^,^85^. In our study, we observed that knockdown of PKCζ significantly reduced endogenous NFκB transcription activity in multiple basal-like breast cancer cells including MDA-MB-231 and HCC-1937 cells. In many cancers, constitutive nuclear NFκB activity has emerged as a hallmark for cancer progression including breast cancer and constitutive NFκB activity often linked to drug resistance and increased cell survival in response to genotoxic stress^86^. Furthermore, constitutive NFκB activity has been reported to induce EMT program in breast cancer cells and development of metastatic disease^83^,^87^. Supportive to these notions, inhibition of NFκB sensitizes many tumor cells to chemotherapeutic drugs^85^. However, constitutive activation of NFκB is regulated though multiple signaling cascades in a context dependent manner^88^ and identification of signaling nodes responsible for constitutive activation of NFκB always provide putative therapeutic target for cancer progression. In fact, NFκB-p65 can repress expression of both E-cadherin and ZO-1 in multiple cell types including mammary epithelial cells^76^,^77^,^89^,^90^ and we observed that depletion of PKCζ significantly upregulated expression of both E-Cadherin and ZO1. Moreover, we also observed repression of both E-cadherin and ZO-1 expression and restoration of invasive potential of PKCζ–depleted cells after ectopic expression of constitutively active NFκB-p65. Thus, it is plausible that PKCζ signaling might be important for constitutive NFκB activity responsible for breast cancer progression and this observation is further supported by our observation that both PKCζ and functionally active phospho-PKCζ expression is significantly higher in aggressive forms of breast cancer.

Our study indicates that downstream to oncogenic PKCζ signaling, NFκB-p65 represses expressions of ZO-1 and E-cadherin in breast cancer cells. Importantly, several EMT-associated transcription regulators such as Snail, Twist 1, ZEB1 and ZEB2 have been reported previously as NFκB-p65 target genes and these transcription factors are known to repress expression of E-cadherin^77^,^89^,^91^. Thus, the repression of ZO-1 and E-cadherin by PKCζ-NFκB-p65 signaling might be mediated indirectly via these transcription regulators. In general, NFκB-p65 is primarily considered a transcriptional activator and S536 phosphorylation of NFκB-p65 has been thought to be associated with transcriptional activation^92^. However, multiple studies also indicated emerging role of NFκB-p65 in transcriptional repression via direct interaction with histone deacetylase (HDAC) co-repressor proteins such as HDAC1^93^,^94^,^95^. Thus, it is tempting to propose that oncogenic PKCζ signaling might be involved in certain posttranslational modification(s) of NFκB-p65 to favor its interaction with HDAC1 or other unknown factors to repress target genes expression such as ZO-1 and E-cadherin. Importantly, the dominant negative S536A mutant of NFκB-p65 has been reported to significantly less effective in repressing gene expression in other system^96^. Consistently, we also observed restoration of ZO-1 and E-cadherin expressions in PKCζ-depleted cells after ectopic expression of dominant active S536E mutant of NFκB-p65. However, we do not know the detailed molecular mechanism yet and future research in this direction will provide new mechanistic information to understand oncogenic PKCζ-NFκB-p65 signaling and to develop potential therapeutic strategy for breast cancer.

Previously, inhibition of PKCζ has been linked to EGFR-induced chemotactic migration of breast cancer cells^54^. In our study, we used MDA-MB-231 cells that express higher level of EGFR^85^. Our observations indicate that PKCζ played a significant role to dictate migration and invasiveness utilizing NFκB-p65 as one of the possible downstream transcription factors. In fact, EGFR induced NFκB activation has been reported to play an inductive role for breast cancer cell migration^85^, however, the downstream mechanism to active NFκB transcription activity is not fully understood. Based on our data, it is tempting to propose that PKCζ might acts as the necessary kinase required for EGFR induced NFκB activation during breast cancer progression and future research in this direction will provide more detailed mechanism of breast cancer growth, invasion, and metastasis.

In summary, our observations indicate an oncogenic PKCζ-NFκB signaling node, which is responsible to regulate intercellular junctional dynamics and facilitates breast cancer cells to achieve invasiveness and metastatic capability. Thus, targeting the oncogenic PKCζ-NFκB signaling node might be beneficial for breast cancer treatment.

## Experimental Procedures

### Cell lines

The MCF-7, MDA-MB-231, MDA-MB-468, and HCC1937 cells were purchased from the American Type Culture Collection. For bioluminescent imaging, the MDA-MB-231-luc cells were generated as described earlier^33^. For knockdown of PKCζ, cells were transduced with lentiviral pGIPZ shRNAmir vector containing short hairpins and GFP reporter (Open biosystem). For isolation of PKCζ-depleted MDA-MB-231-luc cells, transduced dual positive cells were enriched by fluorescence-activated cell sorting using a BD FACSAria™ cell sorter equipped with BD FACSDiva™ software (BD biosciences) with purity of the population more than 85%.

### Lentiviral Particle Generation, Transduction, and Puromycin Selection

Lentiviral particles were prepared as described earlier^33^. The target sequences were summarized in Supplementary Table 1. For transduction, cells were plated in 6-well tissue culture plates (BD Bioscience, Catalog No 353046, 250000 cells per well) and after 24 hours, media were replaced. The cells were transduced with viral particles (at MOI of 10 using the formula [(No. cells X MOI)/Viral Titer] ×1000) in serum free, antibiotic free growth media. Two days after transduction, the media were replaced with normal media supplemented with 2 μg ml^−1^ of puromycin (Fisher Scientific, Catalog No 100552) and the cells continued to grow in the presence of puromycin for successive passages to get nearly 100% GFP positive stable cell lines. For transduction of MDA-MB-231-luc cells, 100000 cells were spin-infected in serum free, antibiotic free growth media containing 8 μg ml^−1^ Polybrene (Sigma-Aldrich) with virus particles at MOI of 25.

### Matrigel Invasion Assay

Matrigel Invasion assays were performed as described earlier^33^. Briefly, Both sides of the transwell filters (8 μM pore size, Costar, Catalog No 3422) were coated with 1 μg ml^−1^ of Matrigel^TM^ (BD Bioscience, Catalog No 354234) at 37 °C (500 μl underside and 200 μl in the topside) for 1 hour. Cells were starved for 24 hours in serum free media and plated in serum free media on the upper chamber of the filter wells (200 μl volume, 50,000 cells per well) and the wells were placed on the top of serum containing complete media (600 μl per well) (i.e. with growth factor in the lower bottom of the transwell filters). After incubation, the cells on the transwells were fixed in 10% formalin, stained with 0.1% crystal violet to take pictures with a Nicon SMZ 1500 Stereo Microscope and quantification performed by counting the number of cells present per unit areas (9 unit areas in each field and three fields/well at magnification 8).

### Wound Closure Assay

Wound closure assays were performed as described previously^33^. Briefly, confluent cells were plated in a 24 well plate (BD Bioscience, Catalog No 353043) and were starved for 24 hours using serum-free media. Wounds were generated on the monolayer of cells and images were at T_0_ before switched to serum containing normal media. Pictures of the same position were taken at appropriate time point and areas of the wounds were measured using ImageJ software.

### Cell Aggregation Assay

Single cells were plated on ultralow attachment 6 well tissue culture plates (Costar, Catalog No 3471) at a density of 10000 cells/ml and cultured in serum-free mammary epithelial basal medium media (Lonza, Walkersville, MD, Catalog No CC-3150) supplemented with 20 ng/mL EGF (Chemicon, Catalog No EA140), 5 μg/mL insulin (Sigma-Aldrich, Catalog No 15500), 1 μg/mL hydrocortisone (Stem Cell Technologies, Catalog No 07904), 20 ng/mL bFGF (Invitrogen; Catalog No 13256), B27 (Invitrogen; Catalog No 17504), 4 μg/mL hePARin (Stem Cell Technologies, Catalog No 07980), 100 IU/mL penicillin, and 100 μg/mL streptomycin (Stem Cell Technologies, Catalog No 07500). Cells were fed every three days by adding additional media to wells. After 7days, diameters and numbers of aggregated cell spheres were measured using Celigo Cytometer (Cyntellect, San Diego, CA).

### NFκB Luciferase Reporter Gene Assay

The *cis*-reporter construct pNFκB luc (Stratagene, Catalog No 219078), containing a luciferase cDNA under a regular TATA box and an enhancer element with five NF-κB binding sites, was transiently transfected into MDA-MB-231, HCC-1937, and BT-20 cells with or without PKCζ depletion using lipofectamine following manufacturer’s recommended protocol. Luciferase activities were measured 48 hours after transfection using Dual-Glo® Luciferase Assay System following manufacturer’s recommended protocol (Promega, Madison, WI, Cat. No. E2920). The experiments were repeated in triplicate.

### Extraction of Nuclear and Cytoplasmic Proteins

Harvested cells were subjected to isolation of nuclear and cytoplasmic protein extraction using a commercially available kit (NE-PER Nuclear and Cytoplasmic Extraction Reagents, Cat # 78833, Thermo Scientific) following manufacturer’s protocol. Separated nuclear and cytoplasmic fractions were analyzed by western blot as described earlier^33^. Histone H3 and GAPDH were used as the markers for nuclear and cytoplasmic fractions, respectively.

### Constitutively Active RelA Expression

The constitutively active NFκB-p65 (RelA) construct T7-RelAS536E^78^ (Addgene, Catalog No 24156) harboring mutation at Serine 536 and substituted with glutamic acid (S536E), was transiently transfected into PKCζ-depleted MDA-MB-231 cells using lipofectamine 2000 following manufacturer’s protocol. Expression of constitutively active RelA was confirmed by western blot analysis 48 hours after transfection and subjected to Matrigel^TM^ Invasion Assay as described earlier.

### RNA Isolation and Quantitative RT-PCR

Total RNA from cells was isolated using RNeasy Mini Kit (Qiagen, Catalog No 74104) using manufacturer’s protocol. Complementary DNA was synthesized as described earlier^33^. Briefly, 1 μg of total RNA and 5:1 mixture of random and oligo(dT) primers were heated at 68 °C for 10 min. This was followed by incubation with moloney murine leukemia virus reverse transcriptase (50 units) (Invitrogen) combined with 10 mM dithiothreitol, RNasin (Promega, Madison, WI), and 0.1 mM dNTPs at 42 °C for 1 hour. Reactions were diluted to a final volume of 100 μl and heat-inactivated at 97 °C for 5 min. 20- μl PCR reactions contained 1 μl of cDNA, 10 μl of 2X SYBR Green Master Mix (Applied Biosystems, Foster City, CA), and 100–300 nM of corresponding primer sets. Primers were listed in Supplementary Table S2. Reactions, lacking reverse transcriptase, were used as control. Product accumulation was monitored by SYBR Green fluorescence using Step-one Plus real time PCR system (Applied Biosystems, Carlsbad, CA). Control reactions using water yielded very low signals. Relative expression levels were determined from a standard curve of serial dilutions of cDNA samples of human universal RNA (Stratagene, Santa Clara, CA, Catalog No 740000) and were normalized to the expression of HPRT1.

### Western Blotting

Whole cell lysates were prepared with a lysis buffer and the western blot analyses were performed as described earlier^33^. The primary antibodies for western blot analysis were listed in Supplementary Table 3. The membranes were stripped by incubating with stripping buffer (50 mM Tris-HCl, pH 6.8, 2% SDS, and 100 mM β-marcaptoethanol) at 50 °C for 30 min followed by washing with TBST 3–4 times (15 minutes each) and reprobed whenever necessary. Quantifications performed by measuring intensities of the bands of interest using ImageJ software.

### Immunofluorescence

Cells were cultured on glass cover slip, washed with PBS and fixed with 4% paraformaldehyde to perform immunofluorescence analysis as described earlier^33^. The primary antibodies and their dilutions for immunofluorescence analysis were listed in Supplementary Table 3.

### Immunohistochemitry (IHC)

Harvested tissues from transplanted mice were fixed in 4% paraformaldehyde at 4 °C overnight and 5 μm thick tissue sections were subjected to immunohistochemistry after deparaffinization at 56 °C for 1 h followed by treatment with 1% hydrogen peroxide for ten minutes. Antigen retrieval were performed using Reveal Decloaker (Biocare Medical, CA, USA) following manufacturer’s protocol and incubated with a blocking buffer followed by primary antibody of interest as listed in Supplementary Table 3. Biotinylated secondary antibodies and an ABC avidin- biotin-DAB detection kit (Vector laboratories, CA, USA) were used for visualization following manufacturer’s protocol. Stained slides were analyzed under Olympus Imaging microscope.

### Animal Studies and Bioluminescent Imaging

All animal work was done in accordance with a protocol approved by the Institutional Animal Care and Use Committee of the University of Kansas Medical Center. Female NOD-SCID NSG mice (Charles River) of 4–6 weeks old were used in xenograft studies for both spontaneous metastasis development and lung metastatic colonization assays. PKCζ knockdown MDA-MB-231-luc cells (shRNA clone 2) were harvested in PBS and subsequently injected into the mammary fat pad or lateral tail vein in a volume of 0.1 ml as described earlier^33^. Following isoflurane-induced anesthesia, mice were imaged for luciferase activity immediately after injection to exclude any that were not successfully xenografted as described earlier^33^. Imaging were performed with a Xenogen IVIS® system coupled to Living Image® acquisition and analysis software version 4.0 (Xenogen) and described earlier^33^.

### Analysis of human primary breast tumor samples by IHC

The tissue microarray slides consist of 55 IDCs (10 ER positive, 10 HER2 positive, 35 TNBC), 10 DCIS, 10 normal breast tissues, and 10 metastatic breast cancers were prepared by the University of Kansas Medical Center Department of Pathology from archival material following IRB approval. Expression levels of both PKCζ and phospho-PKCζ were analyzed by immunohistochemistry. Digital images of the stained slides were taken using Aperio® TMA software and expression of both PKCζ and phospho-PKCζ were analyzed by two independent pathologists in a double-blind fashion. The expression levels (IHC scores) were indicated in a scale of 0 to 3, where 3 indicates highest and 0 indicates lowest expressions. IHC scores 0 to 1 and >1 were considered as low and high expression respectively. Expression level value greater than 1 is considered as high and the significance were calculated by two-way ANOVA with Boneferroni post-test.

### Statistical Analysis

All statistical analyses were performed using GraphPad Prism5 statistical software (GraphPad Software Inc., San Diego, CA). All data are expressed as means ± S.E.M. *P*-values were calculated by two-tailed unpaired Student’s *t* test and one-way or two-way ANOVA with Bonferroni post-test. *P* < 0.05 was considered as significant.

## Additional Information

**How to cite this article**: Paul, A. *et al.* PKCζ Promotes Breast Cancer Invasion by Regulating Expression of E-cadherin and Zonula Occludens-1 (ZO-1) via NFκB-p65. *Sci. Rep.* **5**, 12520; doi: 10.1038/srep12520 (2015).

## Supplementary Material

Supplementary Information
