# Supplementary material for: PKCζ Promotes Breast Cancer Invasion by Regulating Expression of E-cadherin and Zonula Occludens-1 (ZO-1) via NFκB-p65
Source: Sci Rep. 2015 Jul 28;5:12520. doi: 10.1038/srep12520 (PMC4648478; doi:10.1038/srep12520)
Supplement: Supplementary Information [file srep12520-s1.pdf]

SUPPLEMENTARY INFORMATION

TITLE:

PKCζ Promotes Breast Cancer Invasion by Regulating Expression of E-cadherin and Zonula Occludens-1 (ZO-1) via NFκB-p65

RUNNING TITLE: PKCζ-NFκB Signaling in Breast Cancer

AUTHORS:

Arindam Paul[1, 2, 3 *], Marsha Danley[1, 2], Biswarup Saha[2], Ossama Tawfik[1, 2] and Soumen Paul[1, 2,3]

AUTHORS AFFILIATIONS:

[1] The University of Kansas Cancer Center, University of Kansas Medical Center, Kansas City, KS 66160, USA
[2] Department of Pathology and Laboratory Medicine, University of Kansas Medical Center, Kansas City, KS 66160, USA
[3] Institute of Reproductive Health & Regenerative Medicine, University of Kansas Medical Center, Kansas City, KS 66160, USA

[*] Corresponding Author

INVENTORY OF SUPPLEMENTARY INFORMATION

Supplementary Figure S1, related to Figure 1
Supplementary Figure S2, Related to Figure 3
Supplementary Figure S3, Related to Figure 4
Supplementary Figure S4, Related to Figure 6
Supplementary Figure S5, Related to Figure 7
Supplementary Table 1-3, related to Materials and Methods

CONTACT INFORMATION:
Arindam Paul
University of Kansas Medical Center
3901 Rainbow Blvd, Kansas City, Kansas 66160.
Tel: 913-588-7231
Fax: 913-588-7073

Email: apaul2@kumc.edu

Supplementary Figure S1

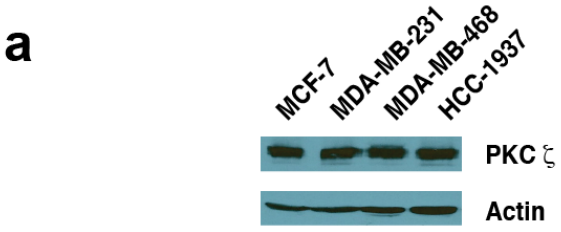

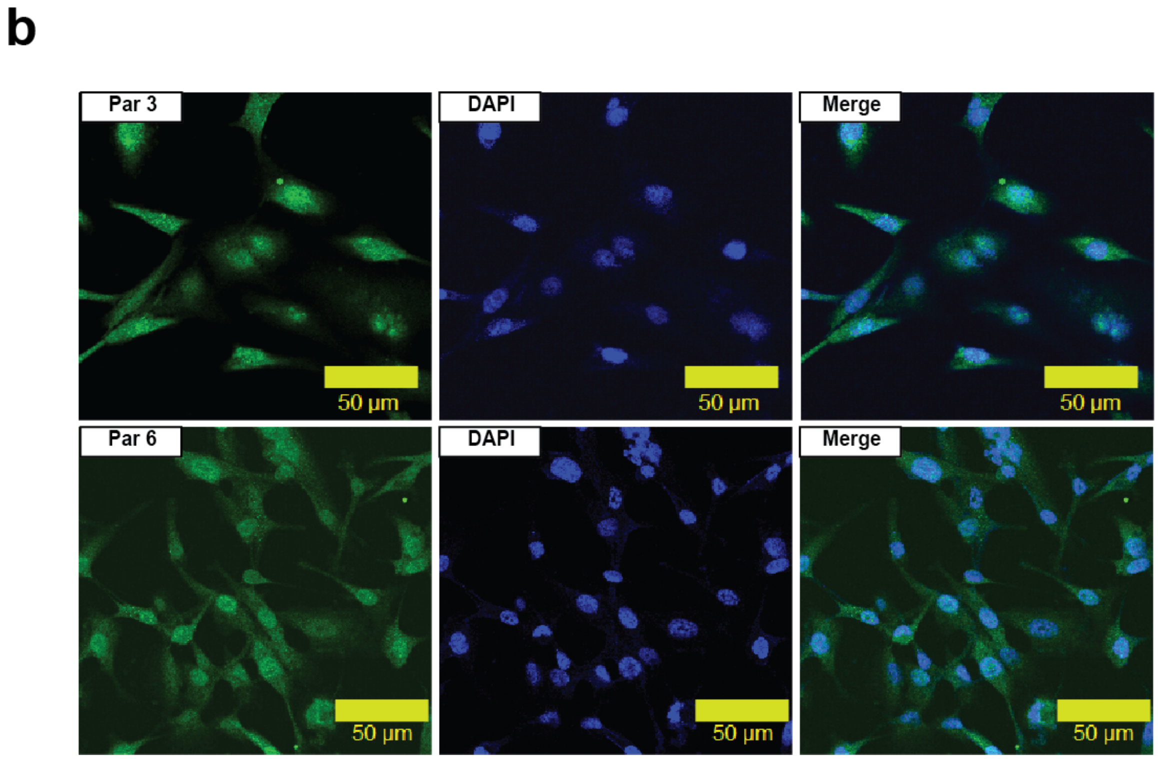

Supplementary Figure S1 (Related to Figure 1): Expression of PKCζ, PAR3, and PAR6 in basal-like breast cancer cells. (a) Western blot analysis in luminal MCF-7 and basal-like MDA-MB-231, MDA-MB-468 and HCC-1937 cells showed no significant difference in the PKCζ expression level. **(b)** MDA-MB-231 cells have no functional PAR polarity complex. Expression of polarity proteins PAR3 and PAR6 in MDA-MB-231 cells showed diffuse staining pattern indicating absence of functional PAR complex at plasma membrane. Scale bar 50 μM.

Supplementary Figure S2

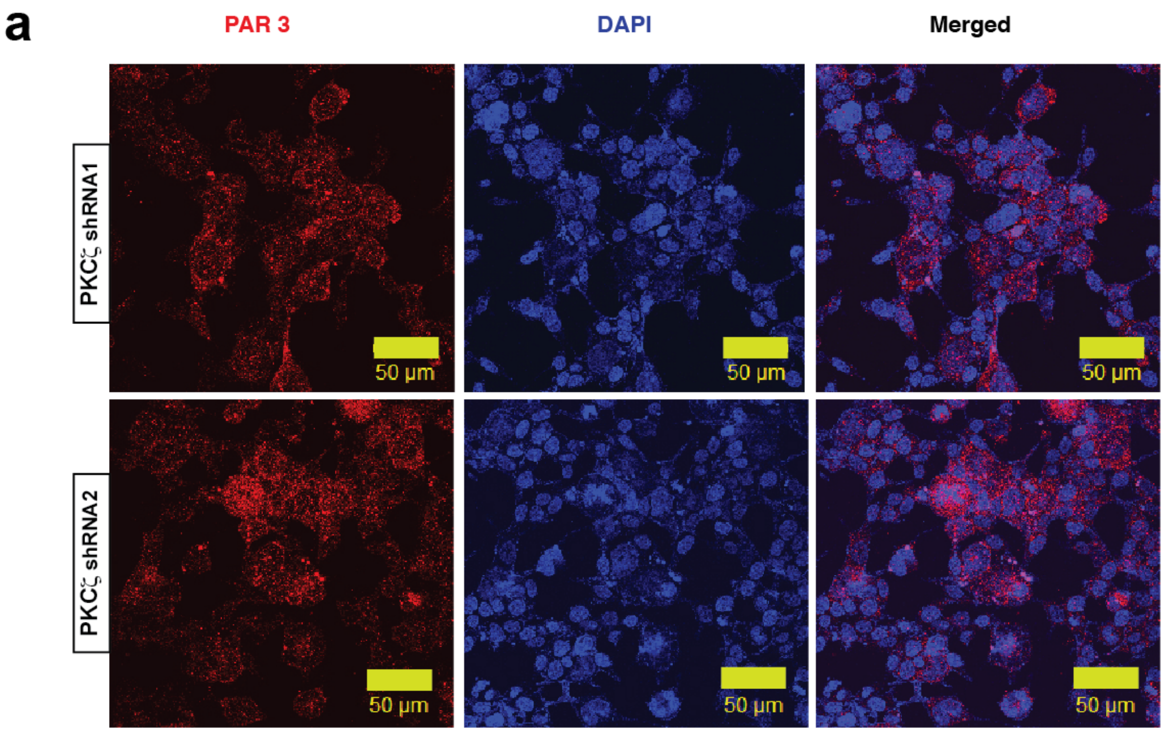

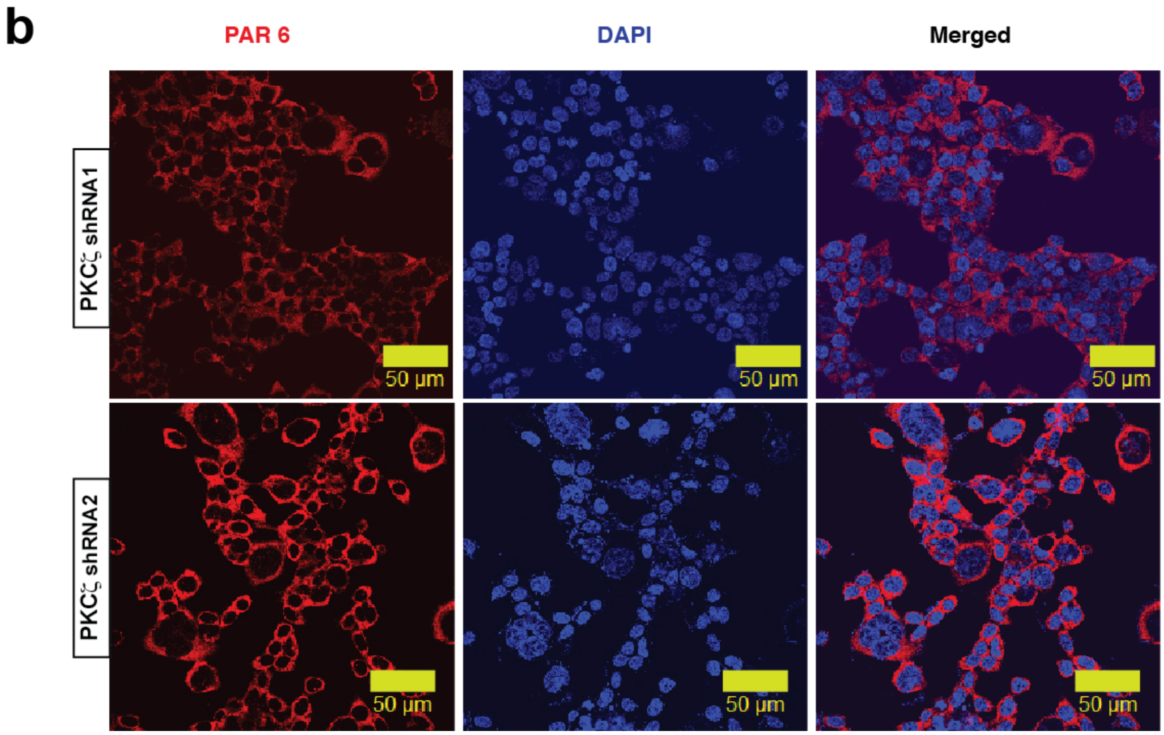

Supplementary Figure S2 (Related to Figure 3): Expression and localization of PAR3 and PAR6 in PKCζ-depleted MDA-MB-231 cells. Expression of polarity proteins PAR3 (**a**) and PAR6 (**b**) in PKCζ-depleted MDA-MB-231 cells also showed absence of functional PAR complex at plasma membrane. Scale bar 50 μM.

Supplementary Figure S3

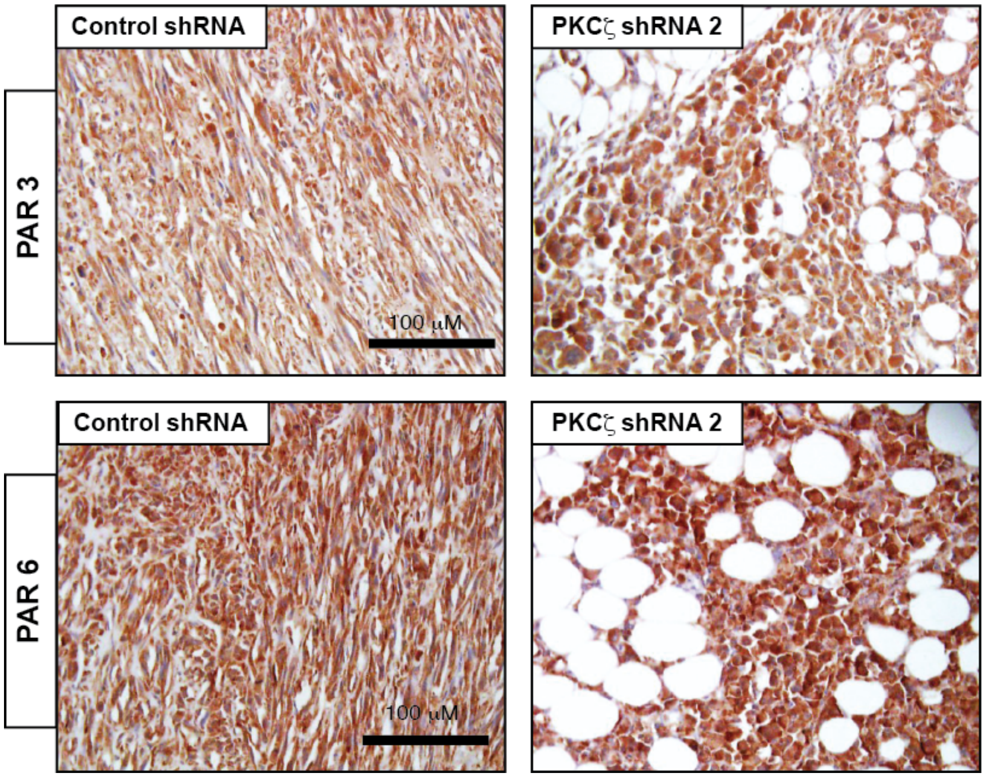

Supplementary Figure S3 (Related to Figure 4): Expression and localization of PAR3 and PAR6 in xenograft tumors. Expression and localization of PAR3 and PAR6 in xenograft tumors formed by MDA-MB-231 cells with and without PKCζ depletion. Scale bar 100 μM.

Supplementary Figure S4

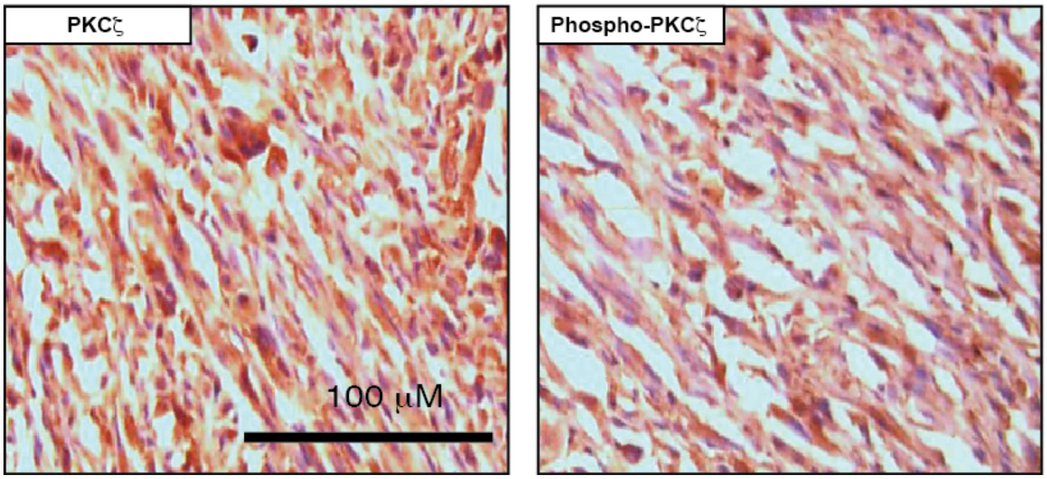

Supplementary Figure S4 (Related to Figure 6): Expression and localization of PKCζ and phospho-PKCζ in xenograft tumors. (**a**) Immunohistochemical analysis of PKCζ and phospho-PKCζ expression and localization in control MDA-MB-231 xenograft tumors.

Supplementary Figure S5

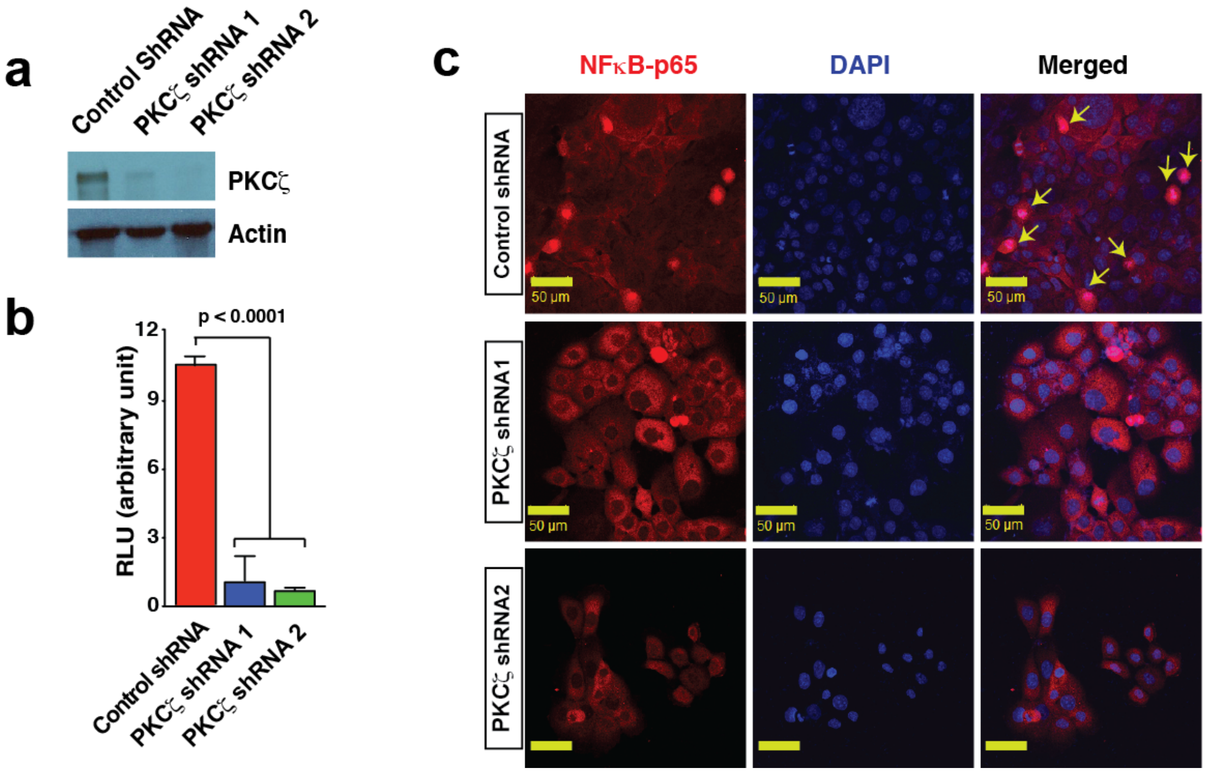

Supplementary Figure S5 (Related to Figure 7): PKCζ-NFκB-p65 Axis also present in basal-like breast cancer cell line HCC-1937. (**a**) Western blot analysis indicating knockdown of PKCζ in HCC-1937 cells. (**b**) NFκB reporter gene assay of HCC-1937 cells with and without PKCζ depletion (n = 3). Results represent means ± S.E.M. P values were calculated using two-tailed unpaired Student's t test. (**c**) Localization of NFκB-p65 in HCC-1937 cells with and without PKCζ depletion. Expression of NFκB-p65 showed in red and nuclear staining showed by DAPI. Scale bar, 50 μM. Yellow arrows indicated nuclear localization of NFκB-p65.

Supplementary Table 1: RNA Interference (shRNA) sequences.

shRNA against gene	Clone Name	Catalog No	Target Sequence (5'–3')
Prkcζ	ShRNA1	V3LHS_641463	CAATGACCAGGAACAACCG
	ShRNA2	V3LHS_641464	CGGTGATGATGTCGAACGG
Control	Control shRNA	RHS 4346	CTTACTCTCGCCCAAGCGAGAG

Supplementary Table 2: List of primers used for Quantitative Real Time-PCR analysis

**Gene symbol**	**Forward (5'–3')**	**Reverse (5'–3')**
CDH1	TGCCCAGAAAATGAAAAAGG	GTGTATGTGGCAATGCGTTC
PRKCZ	ATGACGAGGATATTGACTGGGT	CAGGAGTGTAATCCGACCAGG
ZO-1	ACCAGTAAGTCGTCCTGATCC	TCGGCCAAATCTTCTCACTCC
HPRT1	ACCCTTTCCAAATCCTCAGC	GTTATGGCGACCCGCAG

Supplementary Table 3: List of antibodies used for IHC, IF, and WB

Antibody	Company	Catalog No.	Dilution for IHC	Dilution for IF .	Dilution for WB
PKCζ	Santa Cruz Biotech.	SC-216	1:100	1:400	1:600
p-PKCζ	Santa Cruz Biotech.	SC-12894-R	1:100	1:400	1:600
PKCλ/ι	BD Bioscience	610175	--	---	1:1000
p-PKCλ/ι	Abcam	ab5813	--	---	1:1000
PKCα	Santa Cruz Biotech.	SC-208	---	---	1:600
PKCβI	Santa Cruz Biotech.	SC-209	---	---	1:600
p-PKCβI	Santa Cruz Biotech.	SC-101776	---	---	1:600
PKCδ	Santa Cruz Biotech.	SC-937	---	---	1:600
PKCε	Santa Cruz Biotech.	SC-214	---	---	1:600
PKCγ	Santa Cruz Biotech.	SC-211	---	---	1:600
ZO-1	Cell Signaling	8193	1:200	--	1:1000
ZO-2	Cell Signaling	2847	---	---	1:1000
ZO-3	Cell Signaling	3704	---	---	1:1000
Afadin	Cell Signaling	13531	---	---	1:1000
E-cadherin	Abcam	ab1416	1:100	---	1:500
PAR 3	Millipore	07-330	1:100	1:100	
PAR 6	Abcam	ab49776	1:100	1:100	
NF−κB p65 (RelA)	Cell Signaling	8242	--	1:400	1:1000
Histone H3	Cell Signaling	9715S	---	---	1:3000
GAPDH	Sigma-Aldrich	G8795	---	---	1:25000
β Actin	Sigma	A5441	---	---	1:3000
Ki67	Daco	M 7240	1:200	---	---
